# Experimental and husbandry procedures as potential modifiers of the results of phenotyping tests

**DOI:** 10.1016/j.physbeh.2012.03.026

**Published:** 2012-07-16

**Authors:** Anna-Karin Gerdin, Natalia Igosheva, Laura-Anne Roberson, Ozama Ismail, Natasha Karp, Mark Sanderson, Emma Cambridge, Carl Shannon, David Sunter, Ramiro Ramirez-Solis, James Bussell, Jacqueline K. White

**Affiliations:** Mouse Genetics Project, Wellcome Trust Sanger Institute, Wellcome Trust Genome Campus, Hinxton, Cambridge, CB10 1SA, UK

**Keywords:** Stress response, Husbandry, Systematic large-scale phenotyping, Core temperature, Blood glucose, Radiotelemetry

## Abstract

To maximize the sensitivity of detecting affects of genetic variants in mice, variables have been minimized through the use of inbred mouse lines, by eliminating infectious organisms and controlling environmental variables. However, the impact of standard animal husbandry and experimental procedures on the validity of experimental data is under appreciated. In this study we monitored the impact of these procedures by using parameters that reflect stress and physiological responses to it. Short-term measures included telemetered heart rate and systolic arterial pressure, core body temperature and blood glucose, while longer-term parameters were assessed such as body weight. Male and female C57BL6/NTac mice were subjected to a range of stressors with different perceived severities ranging from repeated blood glucose and core temperature measurement procedures, intra-peritoneal injection and overnight fasting to cage transport and cage changing.

Our studies reveal that common husbandry and experimental procedures significantly influence mouse physiology and behaviour. Systolic arterial pressure, heart rate, locomotor activity, core temperature and blood glucose were elevated in response to a range of experimental procedures. Differences between sexes were evident, female mice displayed more sustained cardiovascular responses and locomotor activity than male mice. These results have important implications for the design and implementation of multiple component experiments where the lasting effects of stress from previous tests may modify the outcomes of subsequent ones.

## Highlights

► Experimental and husbandry procedures affect mouse physiology and behaviour. ► The effects are procedure, parameter and sex dependent. ► A prolonged cage-change interval has no adverse effect on mouse well-being. ► Mice do not habituate to cage changing. ► Successful re-housing of cage-mates after surgical procedures is achievable.

## 1 Introduction

Following the elucidation of the mouse genome sequence, comprehensive functional annotation of the mouse genome is pivotal to understanding the normal function of all genes and the mechanisms through which genetic variants cause disease. As the effort to generate mutant alleles for all mouse genes progresses with pace [1,2], the most facile and informative methods to elucidate phenotypes from knockout mice at large scale and high throughput remain a challenge [3]. The resource of targeted and gene trap alleles in the embryonic stem cell lines generated by members of the International Knockout Mouse Consortium (IKMC) provide a foundation of alleles on a uniform genetic background for large scale phenotyping programmes [4]. Several programmes have been initiated to generate and systematically phenotype the mice from these resources including the Sanger Mouse Genetics Project (MGP), European Mouse Disease Clinic (EUMODIC) and the International Mouse Phenotyping Consortium (IMPC) [5]. The Sanger Mouse Genetics Project will generate, characterise and archive more than 1000 lines of knockout mice over the next 5 years. The phenotyping screens employed are designed to identify genes which perturb the function of the organism via their affect at different levels from small subsets of cells and tissues through to organs and complex physiological systems. A phenotype results from a complex interaction arising from the genetic mutation but influenced by other alleles in the genetic background and the environment. Changes in any one component can influence the phenotype. Mouse geneticists have long appreciated the advantage of controlling for factors which influence phenotype, most notably genetic variation, by use of inbred strains, and limiting exposure to infectious organisms. Environmental factors are also important and their influence on results is under appreciated [6]. In a pivotal paper by Crabbe et al. [7], strain differences and variation between laboratories were highlighted. Since this study was published, further supporting evidence has been published which describes data variability attributed to strain [6,8,9], age, sex [10,11], diet [11], housing [10,12,13] and husbandry practices [10]. To achieve the throughput of alleles required for large scale phenotyping projects it is necessary to minimize the number of animals assayed. It follows that there will be a corresponding loss of sensitivity in the phenotyping assays unless sources of variation are identified and controlled. Accordingly efforts to minimize variation in phenotyping tests is central to the progress and productivity of such projects [6,7,14].

It has been reported that stress from research and husbandry procedures may have an adverse effect on the well-being of laboratory animals [15,16]. Observation on the influence of these stresses have been reported using the rat [16,17], but it is unclear how these results extrapolate to mice, given the differences in mouse and rat physiology and their responses to stress [18,19]. The research community would benefit greatly from understanding the effects of stress in mice, with particular focus on refining working practices to reduce data variability which would reduce the numbers of animals required in an experimental protocol and provide a greater understanding of parameters which could improve mouse welfare.

The current study was conducted to determine the potential impact of stress resulting from common husbandry and experimental procedures on mouse welfare, data quality and reproducibility. Specifically, we examined changes in cardiovascular (CV) parameters, fasted blood glucose (BG) and core temperature (Tc) parameters during BG and Tc measurement procedures, sham intraperitoneal (ip) injection, cage transport, overnight fast and cage changing, the latter two procedures encompassing novel environment as a potential stressor. We report the effect of stress on data quality and highlight that stress is a powerful source of variability which must be considered when standardising protocols and when comparing inter-laboratory data.

## 2 Materials and methods

### 2.1 Animals

The care and use of all mice in this study were in accordance with the UK Home Office regulations, UK Animals (Scientific Procedures) Act of 1986 and were approved by the Wellcome Trust Sanger Institute Ethical Review Committee. C57BL/6NTac mice of both sexes were bred and maintained in a specific pathogen-free unit, room temperature and humidity regulated (21 ± 2 °C; 55 ± 10%), 12/12 h light/dark cycle with lights off at 19:30 h and no twilight period. Mice were housed in individually ventilated cages (IVC) (Techniplast Seal Safe 1284 L) receiving 60 air changes per hour, at a stocking density of 4–5 mice per cage unless otherwise stated below [overall dimensions of caging (L × W × H): 398 × 215 × 187mm, floor area = 530 cm^2^]. Aspen bedding substrate and standard environmental enrichment of nestlet, cardboard tunnel, and three wooden chew blocks were provided. Mice were given water and breeding diet (irradiated A03, SAFE, France) *ad libitum* unless otherwise stated in the methods below.

### 2.2 Experimental procedures

#### 2.2.1 Transmitter implantation and post operative care

One mouse from each cage of 4 mice was selected for radiotelemetry transmitter implantation surgery. In total, 7 female (weighing 30.6 ± 0.8 g before surgery (mean ± SEM)) and 6 male (weighing 36.6 ± 1.6 g before surgery (mean ± SEM)) C57BL/6NTac mice at 17–18 weeks of age underwent surgical implantation of a radiotelemetry transmitter with a weight of 1.4 g (TA11PA-C10, Data Sciences International, USA). Mice were anaesthetized by inhalation of isofluorane and oxygen (induction: isofluorane 2.5%: oxygen 1 L/min; maintenance: isofluorane 1–1.5%: oxygen 0.5–0.7 L/min) and eyes were protected from drying with ointment (Viscotears Liquid Gel, Pharma GmbH, Germany). A midline incision was made through the skin on the ventral side of the neck, the flexible catheter of the radiotelemetry probe was inserted into the left common carotid artery and the transmitter body placed subcutaneously along the right flank. Silk sutures (Mersilk, Ethicon Inc, UK) were used to close the incision and remained in place until termination of the animal. Analgesia was administered (Buprenorphine, Patheon UK Ltd, UK, 0.1 mg/kg s.c.) immediately after anaesthesia. Further doses of buprenorphine were given immediately and 24 h post surgery. Post-operatively, mice were placed in a recovery rack pre-heated to 30 °C for 2 h. When mice were fully ambulant they were housed in their individual cages. In addition to normal food and water, a dietary supplement (Complan, Complan Food Ltd, UK), was given to the implanted mice for 14 days to support recovery and minimise risk of welfare complications and/or loss of test subjects due to poor health. Visual examination of the overall condition of all mice and body weight (BW) checks were performed daily for 14 days post surgery. BW recordings were taken each morning between 8 am and 10 am using Adventurer Pro AV2101 precision balance (Ohaus scales and balances, UK) set to accommodate animal movement. Average weights of implanted and non-implanted mice of both sexes are presented in Supplementary Table 1.

Radiotelemetry measurements were initiated in the fifth week post surgery. Cages were positioned over receivers at all times and transmitters were activated from outside the cage. This enabled measurements to be initiated without cage disturbance. During recording sessions, systolic arterial blood pressure (SAP), heart rate (HR), respiratory rate (derived from blood pressure waveforms) and locomotor activity were monitored using the Dataquest IV system (Data Sciences International, USA). Both scheduled and continuous recording modes were used depending on experimental protocol. Radiotelemetery signals were processed using DataQuest A.R.T. v.4.0 (Data Sciences International, USA).

#### 2.2.2 Re-grouping

The implanted mice were re-grouped with their cage-mates 3 days after surgery in accordance with the recommendations of the BVAAWF/FRAME/RSPCA/UFAW Joint working group [20] and in-house observations. The implanted mice exhibited no overt signs of sickness or distress and wounds were sealed, dry and clean at the time of re-grouping. We implemented the following strategy to prevent or minimise aggressive behaviour towards cage-mates. Upon removal of the mouse selected for surgery, all cage-mates were also individually housed into clean cages but with the addition of a small amount of soiled bedding from their original home cage. Seven days prior to separation, additional nesting material was given so that ample was available for transfer into individual cages and additional environmental enrichment provided to minimise competition upon re-grouping. During separation, transfer of aspen bedding substrate between individual cages was performed daily. Soiled, group home cages were stored unventilated and used to re-house mice after separation. Animals were re-housed with those they had been housed with previously and were rubbed with soiled bedding from all other animals before being added to the group. From this point forward all non-implanted mice also had access to Complan supplement. Cages were stored unventilated for 60 min after re-grouping to allow accumulation of odours.

Over a 10-week period, both implanted mice and their cage-mates underwent several potentially stressful experimental and husbandry procedures frequently encountered in animal units, in the order described in Table 1.

#### 2.2.3 Blood glucose measurement

Animals at 21–22 weeks of age were fasted overnight (up to 16 h) prior to BG measurement procedures, by transferring mice to a clean cage base with clean nesting material and a small amount of soiled bedding and environmental enrichment from their old cage. The change of cage and bedding obviated the possibility that mice may access spilled food. Water remained freely available throughout the entire fasting period. Food was returned following collection of the final blood sample at T_120_, extending the fasting duration to a maximum of 18 h. A drop of blood was obtained from unrestrained mice by nicking the tail tip with a blade. Measurements were taken using a handheld blood glucose meter (Accu-chek Aviva, Roche Diagnostics, UK), beginning between 9 am and 10 am for all test mice. All BG measurements were completed by 12noon.

#### 2.2.4 Core temperature measurement

Rectal Tc measurements were taken on unrestrained mice at 22–23 weeks of age using a DH-5 Monitoring thermometer with associated RET-3 rectal mouse-specific probe (Viking Scientific, USA).

During BG and Tc sampling procedures, the mouse to be measured was identified by earmark and moved from the cage base onto the cage grid, the mouse remaining within range for the DSI receiver to record all responses, and the measurement taken. When BG or Tc values were obtained the mouse was returned to the cage base and the next mouse identified and tested in the same way. Implanted mice were tested first, followed by cage-mates. The order in which mice were removed from group housing and tested was recorded and kept consistent when a repeat measurement was taken. BG and Tc were measured at two time points 120 min apart (T_0_ and T_120_) and cages were undisturbed between measurements. CV parameters were recorded continuously from 20 min prior to the initial BG or Tc measurement until 20 min after the repeat measurement.

#### 2.2.5 Cage change and ammonia concentration

Cage change procedure and ammonia levels were assessed over a 4 week interval commencing when the mice were 23–24 weeks of age. Prior to this testing, cage changes were routinely performed at fortnightly intervals i.e. mice had undergone 11–13 cage changes. During movement of mice to a clean cage both cages were placed side by side on a DSI receiver and the implanted mouse was transferred to the new cage first followed by cage-mates. Clean aspen bedding substrate and nesting material were provided. Environmental enrichment and a small amount of bedding from the soiled cage were transferred. CV parameters were recorded continuously from 24 h prior to cage clean until 48 h after.

Cage changing was performed with either a 7 or 14 day interval and ammonia concentration measured in triplicate in each unpopulated soiled cage. Ammonia concentrations were measured using ammonia specific diffusion tubes and a manual pump [MSA NH3detector tube (range 0–200 ppm), Gas-Tester™II H Detector Tube Pump; Ribble Enviro, UK]. To obtain readings that reflected concentrations within undisturbed cages, soiled cages were left unventilated for 30 min prior to measurement and the tube was inserted through the water bottle aperture and held approximately 3 cm above the bedding at the level of an adult mouse head.

#### 2.2.6 Overnight fast

Mice aged between 27 and 28 weeks were assessed for response to a 16 h period of food deprivation. Food was removed and mice were transferred to a new cage base as described above, 2 h before the onset of dark phase. During movement of mice from old cage to new cage, both cages were placed side by side on a DSI receiver and the implanted mouse was transferred to the new cage first followed by cage-mates. The non-fasted control group was present in the room during the procedure, but their cages were not disturbed. CV parameters were recorded continuously in fasted and non-fasted mice 20 min prior to, and during cage changing and overnight fasting period. Food was re-introduced to cages upon completion of the fasting period.

During a follow-up study additional time points and experimental procedures were measured on mice without radiotelemetric implants that had not been part of the radiotelemetric study. As the magnitude of response to sampling methods was similar in male and female mice, further work was performed only on males to reduce the number of animals used. Non-implanted male mice were distributed over 16 groups of 10 mice housed 5 per cage; each mouse was only a part of one experimental group (Table 2). Mice undergoing BG measurement were tested at 13 weeks of age whilst mice undergoing Tc measurement were tested at 14 weeks of age.

#### 2.2.7 Time course of BG and Tc responses

To gain better insight into the time-course of BG and Tc responses, the BG and Tc methods described above were used to test mice at baseline (T_0_) and a second time-point, either 15 min (T_15_), 30 min (T_30_) or 60 min (T_60_) after the initial measurement. Later time points were sampled by gentle manipulation of the tail; a second incision was not required.

#### 2.2.8 Intraperitoneal injection

BW was captured immediately prior to measurement of BC and Tc. Sham solution (saline, 0.1 ml/10 g body weight) was administered via ip injection then BG and Tc were re-tested at T_15_.

#### 2.2.9 Cage transport

Cages of group housed mice were moved between rooms on a wheeled trolley and stored on a mobile ventilated rack during testing. BG and Tc were taken from one group immediately following transport of cages and from a second group 60 min after transport of cages.

#### 2.2.10 Individual housing

Group housed mice were transferred individually to a clean cage base with aspen bedding substrate and one paper tissue for nesting material. BG and Tc were taken from one group immediately following individual housing and from a second group 60 min after individual housing.

### 2.3 Experimental design and statistical methods

For all experiments detailed below, the experimental unit was an individual mouse and factors thought to affect the variables being measured were standardised throughout the experiment. The one exception occurred for the sex comparisons of radio-telemetric data as the experiments using female mice were performed 11 weeks prior to those performed using male mice. As females are the more docile sex, they were studied first, as this allowed us to assess the severity of the response to post-surgery re-grouping before repeating the experiments using male mice. Although both sexes were investigated separately, any possible age effect was controlled for by performing the same experiments at the same age for both sexes.

Repeat measure designs were used when studying radiotelemetric data in response to a potential stressor applied to implanted mice (*n* = 6 females, 7 males). On the basis of the results of the pilot preliminary study for telemetric recording of blood pressure, at least 6 animals were required to achieve 80% power to detect a 10 mm Hg difference, with a probability of *p* < 0.05. The baseline data collected over the time course before the application of a stressor formed the internal control for each animal. A two-way repeat measure ANOVA was used to examine the effect of fasting and time on the variables monitored. Changes of SAP, HR and locomotor activity during overnight fast were expressed as delta, where delta is the difference between fasted and non-fasted measurements for the same group of animals. The use of delta allowed a comparison across sexes as each animal formed its own internal control. A two-way repeat measure ANOVA was used to assess for significant effect of sex and time. A one-way repeat measure ANOVA was used to examine the effect of procedures (BG and Tc measurement procedure, and cage change) with time and each sex statistically examined independently.

For the study of BW changes in response to the implanting procedure, a mixed design repeated measures ANOVA was used. Analysis by ANOVA on the delta values calculated by subtracting post surgery weight (with subtraction of 1.4 g attributed to the implanted transmitter) from pre-surgery weight allowed a comparison across sexes as each animal formed its own internal control. ANOVA's were followed by post hoc Dunnets tests. All CV data is presented as mean ± Standard Error of the Mean (SEM). Differences were considered significant when *p* < 0.05.

Analysis of BG and Tc measurements obtained during radiotelemetry recordings was performed using a general linear model with date treated as a random block effect, and gender and implant as fixed factors. Time points were compared using a paired *t*-test. Differences were considered significant when *p* < 0.05.

Further studies to examine BG and Tc changes in response to a potential stressor applied to non-implanted male mice used a fully randomised experiment format to compare control and treated animals (*n* = 10 per group). Experiments to investigate the time-course of BG or Tc responses used a repeat measure format where later measurements were compared with measurements taken from the same group of animals at an earlier time point. All BG and Tc were examined using a one-way ANOVA followed by Tukey HSD post hoc testing. Differences were considered significant when *p* < 0.05.

To compare ammonia levels between cleaning regimes, a fully randomised format was used to compare the ammonia level at one week vs. two weeks between cage cleaning. A two tailed, homoscedastic *t*-test (*n* = 6 female, 7 male) was used to detect differences in ammonia concentrations. For this experiment, the experimental unit was an IVC cage previously used to group house 4 female or 4 male mice.

In all cases, the appropriateness of the statistical test was checked using the Levene's test for equality of variances, and the Kolmogorov–Smirnov test to assess the normality assumption. Statistical analyses were performed using Statistica version 5.0 for windows (Statsoft Inc., USA) and GraphPad Prizm v4.

## 3 Results

### 3.1 Effect of 3 day separation and re-housing on male and female mouse body weight

**Fig. 1:**
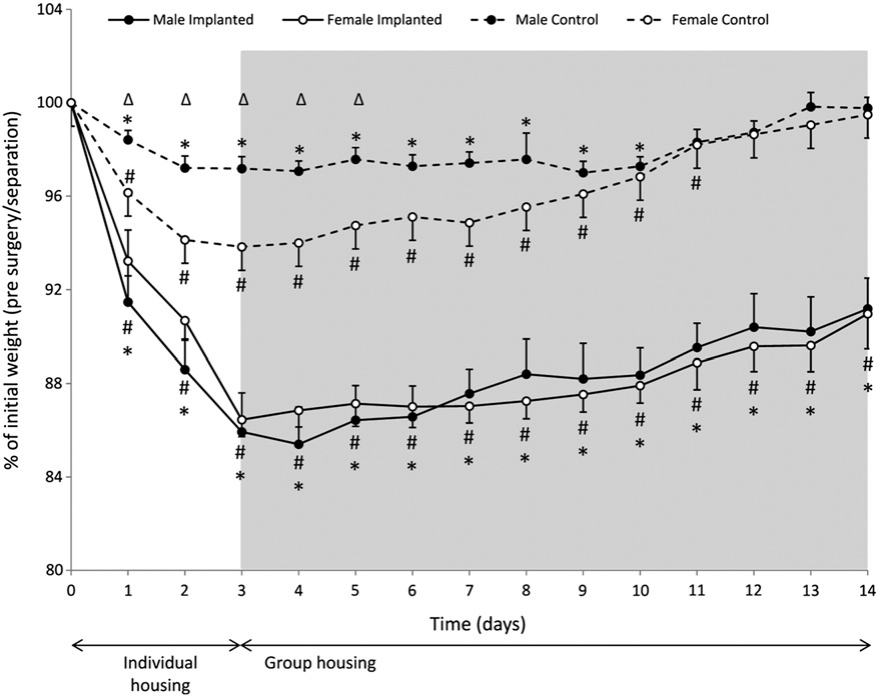
Body weight of male (implanted, *n* = 6; controls, *n* = 18) and female (implanted, *n* = 7; controls, *n* = 21) mice recorded after separation and re-grouping. Data are expressed as percentage change from pre-surgery or pre-separation weight (day 0). Post surgery weights were adjusted by subtraction of 1.4 g to account for the weight of the implanted transmitter. Shaded area indicates days after re-grouping. * – *p* < 0.001–0.0001 vs. Day 0 (males); # – *p* < 0.001–0.0001 vs. Day 0 (females); ∆ – *p* < 0.001 non-implanted males vs. non-implanted females.

In non-implanted mice, 3 day separation and re-housing resulted in significantly decreased BW when compared to pre-separation BW (*p* < 0.001) (Fig. 1 and Supplementary Table 1). This effect was more profound in females (*p* < 0.001) and lasted longer in females (11 days) when compared with males (10 days). Sex had an effect in non-implanted animals with non-implanted females showing a significantly reduced BW during the first 5 days when compared to non-implanted males. Implanted mice of both sexes showed a persistent BW reduction during the first three days post surgery when compared to pre-operation BW (*p* < 0.0001). Sex had no significant effect on this parameter in implanted animals. Although a gradual BW gain was seen from day 5 post surgery, BW remained significantly reduced in implanted mice throughout the 14 day post operative period in comparison to the pre-operation weights. At all time points implanted mice showed significantly more weight loss than non-implanted mice of the same sex (*p* < 0.0001).

### 3.2 Effect of common procedures on cardiovascular parameters in implanted male and female mice

#### 3.2.1 Effect of BG and Tc measurement procedures

**Fig. 2:**
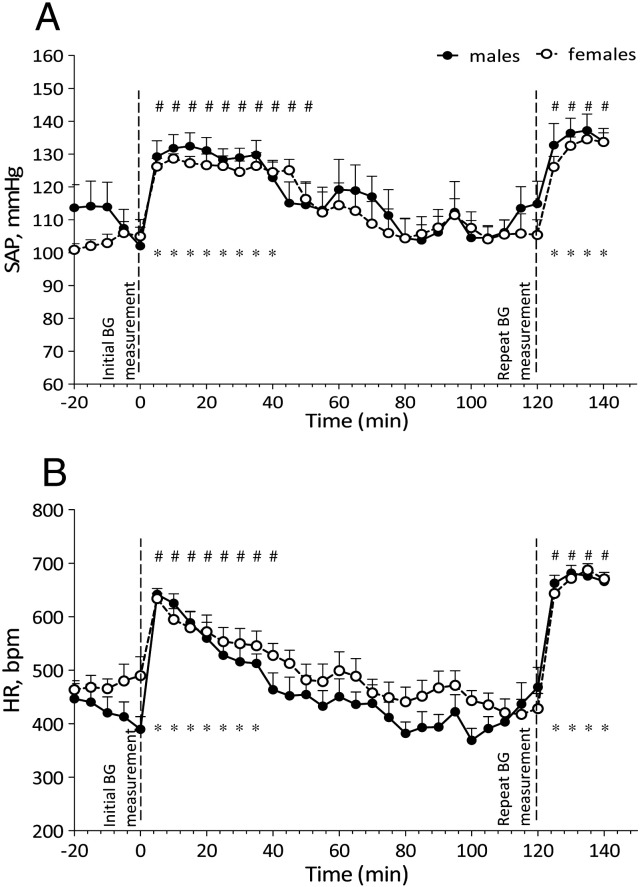
Changes in SAP (A) and HR (B) of implanted male (*n* = 6) and female (*n* = 7) mice during repeat BG measurement procedures following a 16 h overnight fast. Data are expressed as mean + S.E.M. * – *p* < 0.001 vs. baseline (males); # – *p* < 0.05 vs. baseline (females). Baseline is representative of 5 minute averages over the 20 minute time course prior to initial BG measurement procedure.

**Fig. 3:**
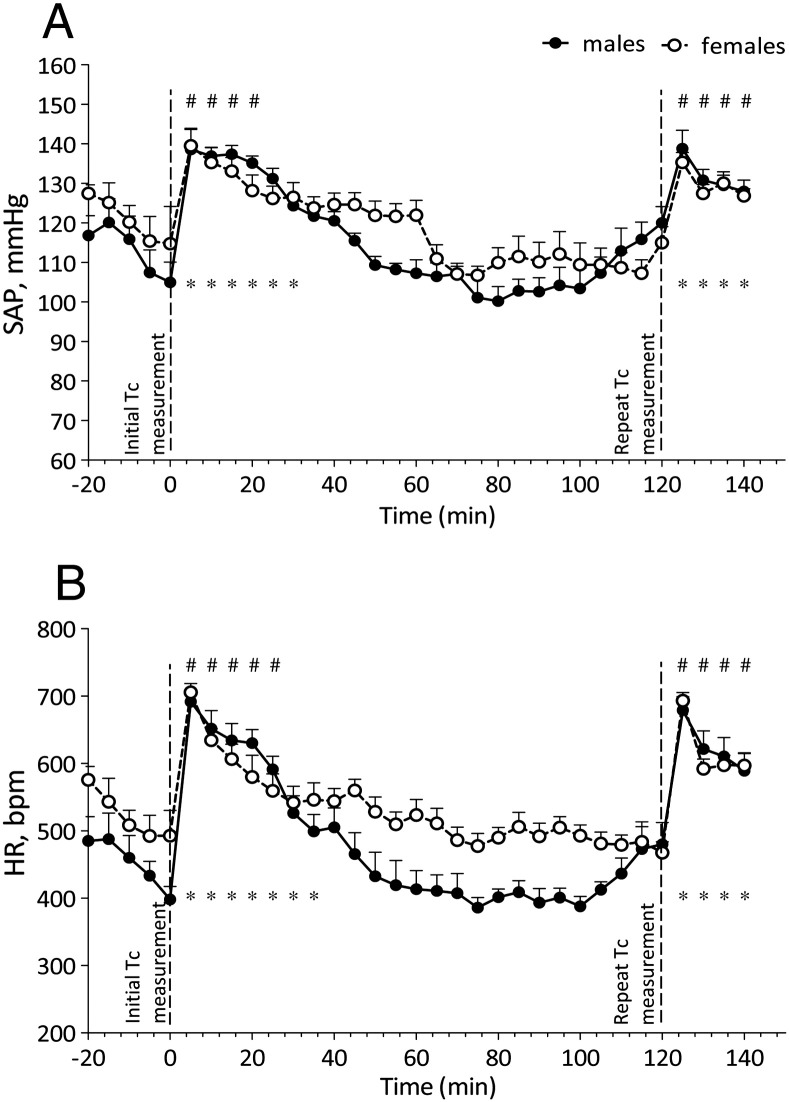
Changes in SAP (A) and HR (B) of implanted male (*n* = 6) and female (*n* = 7) mice during repeat Tc measurement procedures. Data are expressed as mean + S.E.M; * – *p* < 0.001 vs. baseline males; # – *p* < 0.001 vs. baseline females. Baseline is representative of 5 minute averages over the 20 minute time course prior to initial Tc measurement procedure.

The effect of BG and Tc measurement procedures are shown in Figs. 2 and 3, respectively. Both measurement procedures induced a significant and immediate rise in SAP and HR in both sexes when compared with baseline CV recordings (BG males – SAP and HR *p* < 0.001; BG females – SAP *p* < 0.05, HR *p* < 0.001; Tc males and females – SAP and HR *p* < 0.001). The effect of BG measurement lasted longer in females than in males (SAP – 50 min vs. 40 min; HR – 40 min vs. 35 min) whereas the opposite was true after Tc measurement (SAP – 30 min vs. 17 min; HR – 35 min vs. 25 min).

The repeat BG and Tc measurement, 2 h after the initial measurement, also resulted in a significant and immediate rise in SAP and HR in both sexes when compared with baseline CV recordings (BG and Tc males and females – SAP and HR *p* < 0.001). This effect was recorded for 20 min following the sampling procedures. Repeat BG or Tc measurements did not reveal any significant differences between sexes in the magnitude of SAP and HR increase, nor did the magnitude significantly differ between initial and repeat measurements. In both sexes a 120 min interval between BG and Tc measurements was sufficient for SAP and HR to return to baseline levels, baseline being notably lower during BG testing when compared to Tc testing. This difference in baseline can be attributed to the fasted state of mice prior to BG measurement but not prior to Tc measurement and is described further in section 3.2.3. In both sexes a 120 min interval was also sufficient for BG and Tc readings of implanted mice to return to baseline levels (BG males – T_0_ = 4.1 mM, T_120_ = 4.3 mM; BG females – T_0_ = 3.0 mM, T_120_ = 2.9 mM; Tc males – T_0_ = 36.2 °C, T_120_ = 36.0 °C; Tc females – T_0_ = 36.6 °C, T_120_ = 36.5 °C). There was no significant difference in BG or Tc readings between implanted and control animals and no difference between sexes.

#### 3.2.2 Effect of a cage change procedure and frequency

**Supplementary Fig. 1:**
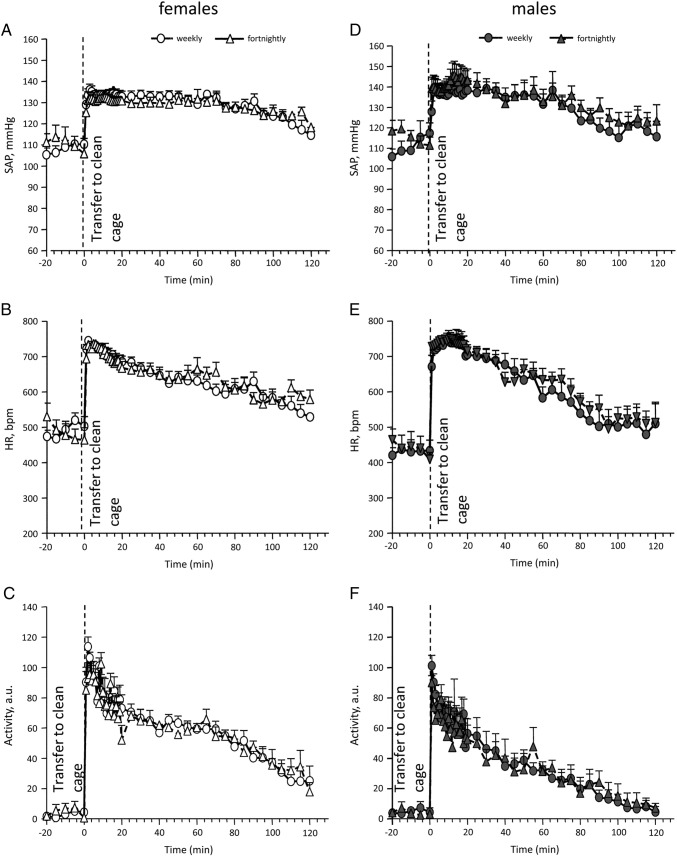
Changes in SAP (A, D), HR (B, E) and locomotor activity in arbitrary units (C, F) of implanted female (*n* = 7) and male (*n* = 6) C57BL/6NTac mice during weekly and fortnightly cage changing. Data are expressed as mean + S.E.M.

**Fig. 4:**
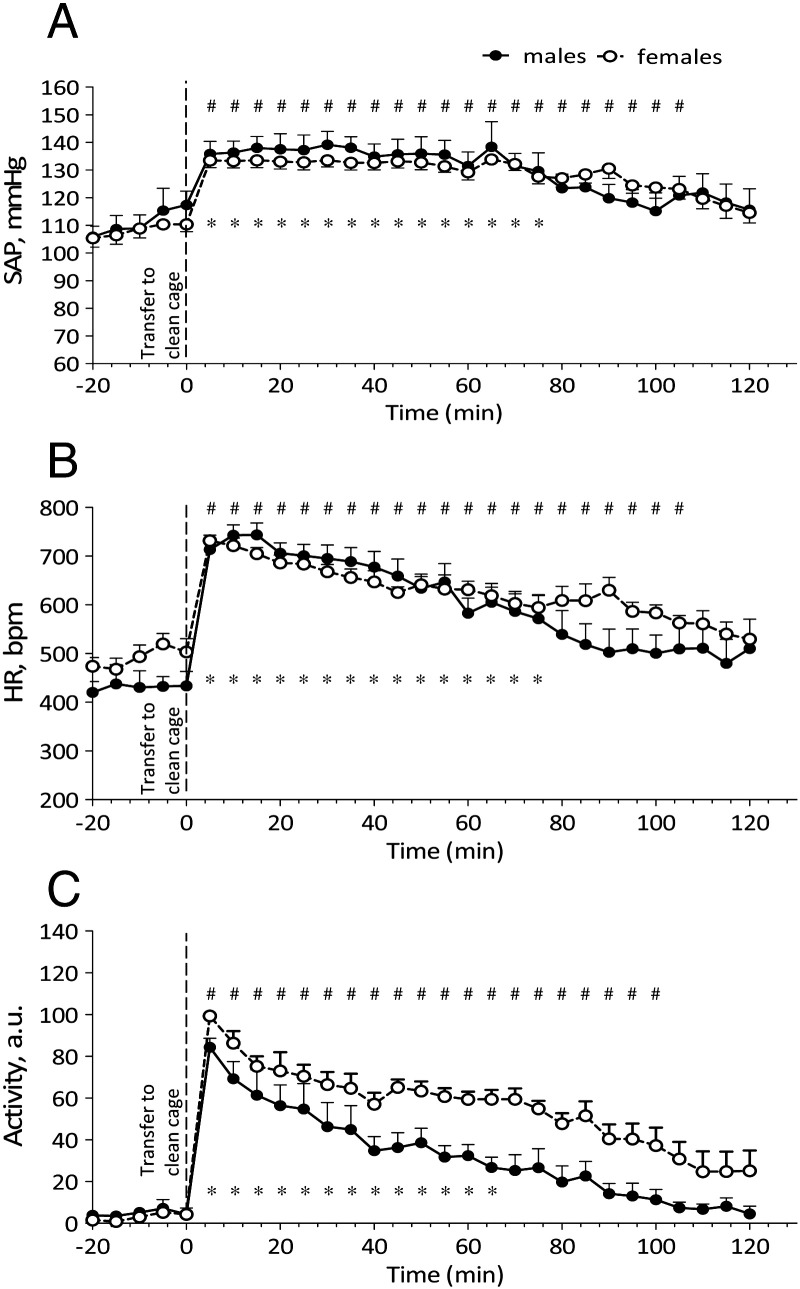
Changes in SAP (A), HR (B) and locomotor activity in arbitrary units (C) of implanted male (*n* = 6) and female (*n* = 7) mice during cage changing. Data are expressed as mean + S.E.M; * – *p* < 0.001 vs. baseline males; # – *p* < 0.001 vs. baseline females. Baseline is representative of 5 minute averages over the 20 minute time course prior to transfer to a clean cage.

Cage change procedure had a significant effect on SAP, HR and locomotor activity (Fig. 4A, B, C) in both sexes when compared with baseline (males and females – SAP and HR *p* < 0.001; locomotor activity *p* < 0.0001). Females showed a more prolonged hypertension and tachycardia following cage change in comparison with males (105 min vs. 75 min). Locomotor activity increase was also more significant (*p* < 0.05) and prolonged (100 min vs. 65 min) in females when compared with males. Cage change frequency (weekly vs. fortnightly) did not significantly influence SAP or HR responses to cage change procedure in either sex (Supplementary Fig. 1).

**Supplementary Fig. 2:**
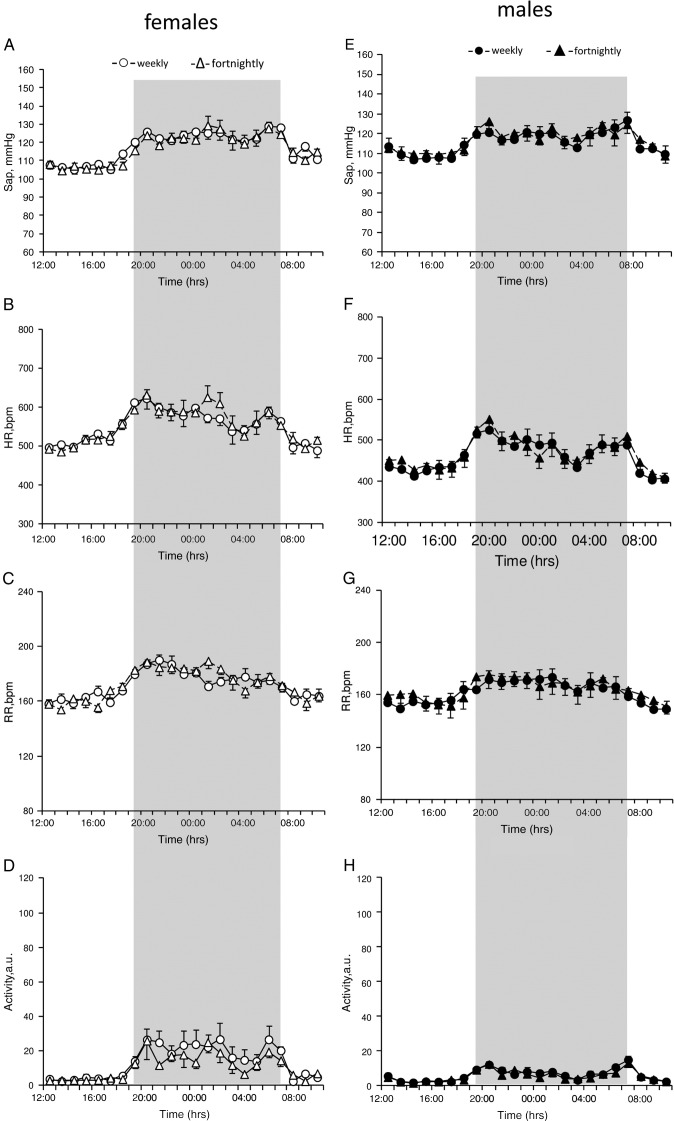
Baseline recordings for 24 h prior to cage clean of SAP (A, E), HR (B, F), respiratory rate (C, G) and locomotor activity in arbitrary units (D, H) in implanted females (*n* = 7) and males (*n* = 6) C57BL/6NTac mice housed in weekly and fortnightly changed cages. Shaded area indicates dark phase room conditions. Data are expressed as mean ± S.E.M.

Ammonia concentrations were higher for both sexes in cages cleaned every two weeks (males – 89 ppm ± 8.43; females – 96.4 ppm ± 10.10) when compared with cages cleaned weekly (males – 74 ppm ± 5.25; females – 57.6 ppm ± 5.86). This increase was significant in females (*p* = 0.0061) but not in males. Despite exposure to a higher ammonia level, basal CV parameters, locomotor activity and respiratory rate of mice housed in cages changed every two weeks did not differ from those housed in weekly changed cages (Supplementary Fig. 2).

#### 3.2.3 Effect of 16 h overnight fasting procedure

**Fig. 5:**
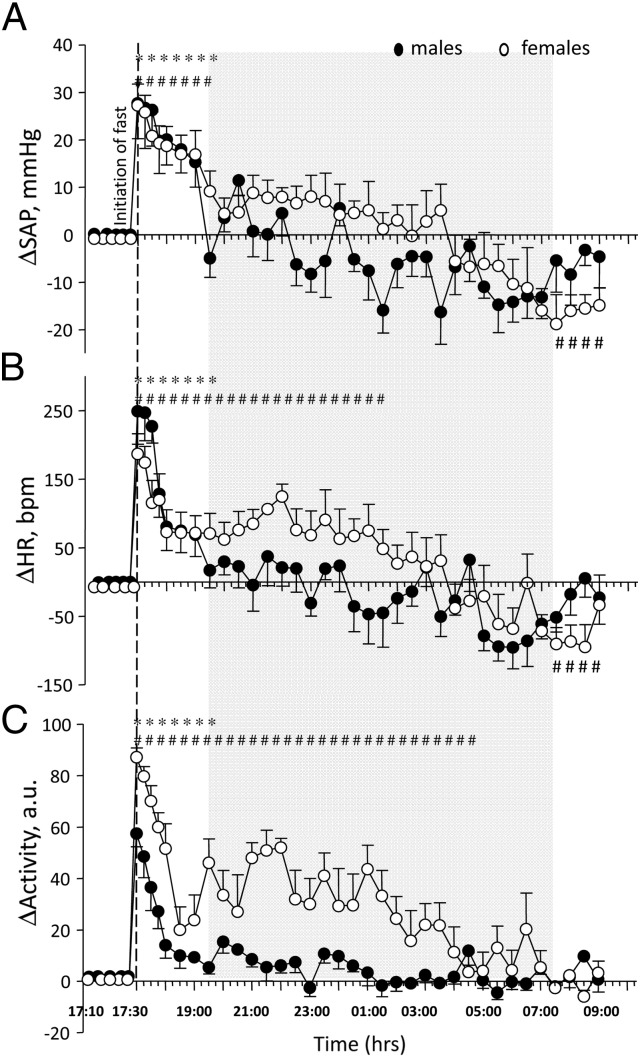
Change in SAP (A), HR (B) and locomotor activity in arbitrary units (C) of implanted male (*n* = 6) and female (*n* = 7) mice recorded during overnight fasting. To begin the 16 h period of food deprivation, food was removed and mice were transferred to a new cage base 2 h before the onset of dark phase. Changes of SAP, HR and locomotor during overnight fast were expressed as delta, where delta is the difference between fasted and non-fasted measurements for the same group of animals. Shaded area indicates dark phase room conditions. Data are expressed as mean ± S.E.M; * – *p* < 0.001 vs. baseline males; # – *p* < 0.001 vs. baseline females. Baseline is representative of 5 minute averages over the 20 minute time course prior to initiation of fast.

An overnight 16 h fasting procedure, beginning at 17:30 h, had a significant effect on both sexes' SAP, HR and locomotor activity when compared with non-fasted control groups (Fig. 5). In females, a marked tachycardia (*p* < 0.0001) and increased locomotor activity (*p* < 0.0001) were evident for a prolonged period (HR – 8 h; locomotor activity – 11 h) (Fig. 5B, C), whereas SAP was increased (*p* < 0.0001) only during the initial light phase (2 h) (Fig. 5A). In males, increases in SAP (*p* < 0.01), HR (*p* < 0.001) and locomotor activity (*p* < 0.001) were also observed but the duration of HR and locomotor activity responses were less than those seen in females (HR – 2 h; locomotor activity – 2 h) (Fig. 5B, C). The magnitude of the increase of locomotor activity was also less profound in males when compared with females (*p* < 0.001) (Fig. 5C). Towards the end of the fasting period (for 2 h after the onset of the light phase) fasted females exhibited a significant fall in HR (*p* < 0.01) and SAP (*p* < 0.01) in comparison to the non-fasted control group whereas such responses were not evident in males.

### 3.3 Effect of a variety of common procedures on BG and Tc in non-implanted male mice

#### 3.3.1 Effect of tail tip blood sampling procedure on BG and Tc in non-implanted mice

**Fig. 6:**
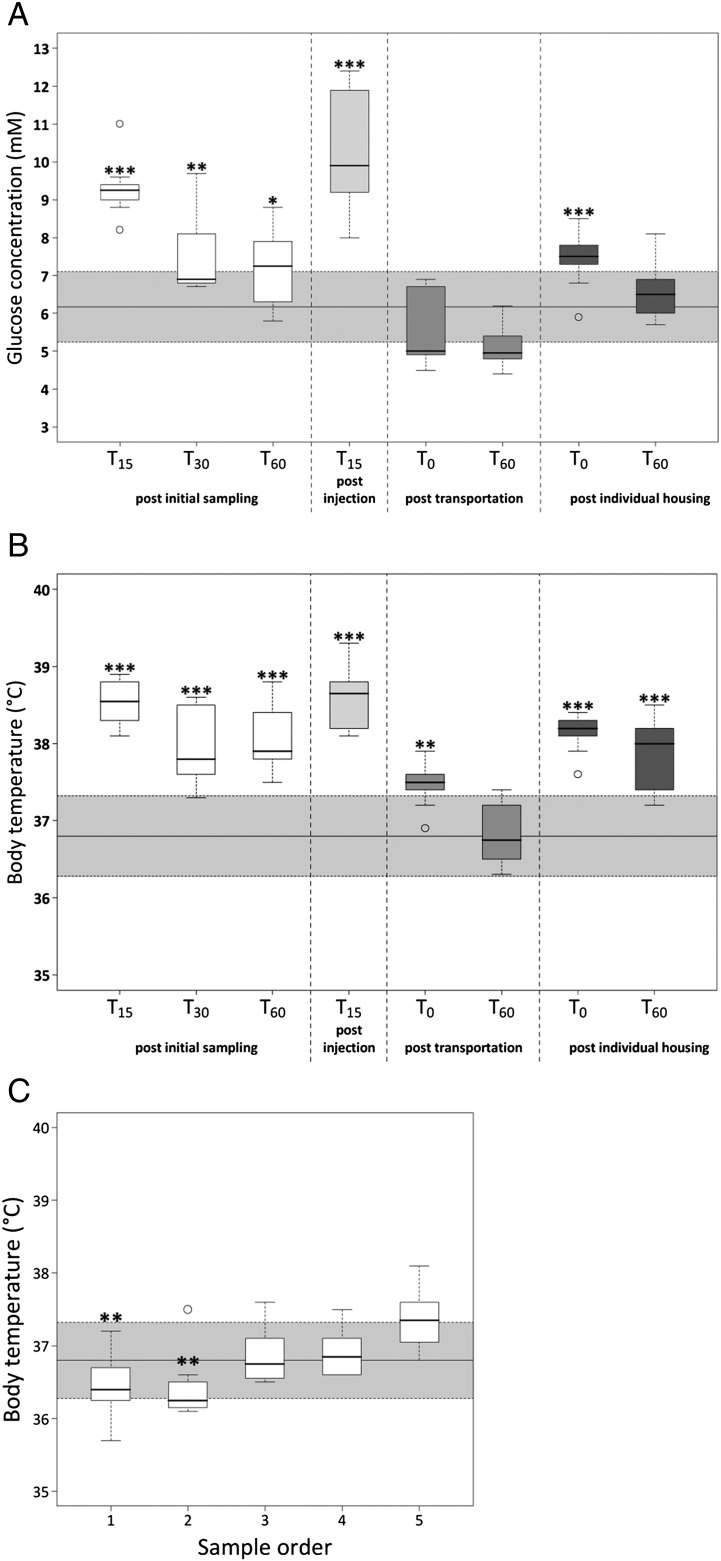
Effect of blood sampling procedure (assessed 15, 30 and 60 minutes after the initial procedure), intraperitoneal injection, cage transportation and individual housing on BG (A) and Tc (B) of non-implanted mice (*n* = 10 per test group). Shaded area represents T_0_ baseline mean (*n* = 39) ± 1SD. Effect of sample order (C) on Tc of non-implanted male mice (*n* = 8 in each test group). Median, 25th and 75th percentile (box) and the lowest and highest data points still within 1.5× the interquartile range (whiskers) for each of the test groups are shown. Data points falling outside the 1.5× IQR are considered outliers and are represented with an open circle. * – *p* < 0.05, ** – *p* < 0.001, *** – *p* < 0.0001.

There was a significant elevation in both BG and Tc at 15 (BG and Tc – *p* < 0.0001), 30 (BG – *p* < 0.001; Tc – *p* < 0.0001) and 60 min (BG – *p* < 0.05; Tc – *p* < 0.0001) after the initial tail tip blood sampling procedure when compared with control, T_0_ measurements (Fig. 6A, B).

#### 3.3.2 Effect of intraperitoneal injection

Sham saline injection resulted in a significant increase in BG (*p* < 0.0001) and Tc (*p* < 0.0001) when compared to control, T_0_ measurements (Fig. 6A, B). Interestingly, the magnitude of this response was not different from that observed 15 min after the initial tail tip blood sampling procedure alone.

#### 3.3.3 Effect of cage transport

Cage transport to the procedure room had no effect on BG but was shown to significantly increase Tc (*p* < 0.001) when compared with non-transported controls. One hour of acclimatization following cage transport, allowed basal Tc to be restored (Fig. 6A, B).

#### 3.3.4 Effect of individual housing

Individual housing was associated with a marked increase in BG (*p* < 0.0001) and Tc (*p* < 0.0001) when compared with group housed controls. One hour of acclimatization period, following individual housing, allowed basal BG to be restored but Tc remained significantly elevated (*p* < 0.0001) (Fig. 6A, B).

#### 3.3.5 Effect of sampling order on BG and Tc in male C57BL/6NTac mice

Sampling order had no effect on BG concentrations but did result in a gradual increase in body temperature on removal of each mouse, culminating in a significant raise in Tc (*p* < 0.001) in mouse 5 compared with mouse 1 and 2, Fig. 6C.

## 4 Discussion

Here we describe stress-like responses in mice caused by routine husbandry and experimental procedures, and focus on how such practices affect mouse physiology, behaviour and data quality. We aimed to identify sources and manifestations of stress, reduce stress through refinements to working practices and utilise the effects of stress to our advantage when the cause is unavoidable.

In this study we have demonstrated that BG and Tc measurements activate the cardiovascular system which declines between tests but is re-activated to the same level by a subsequent measurement. We show that an overnight fast was associated with significant and sex-specific changes in CV and locomotor activities. The procedure-induced changes observed in behavioural and physiological parameters were more prevalent in females than in males. We have also extended and confirmed previously published observations on the impact of BG and Tc measurement [18,21], ip sham injections [22,23], cage transport [18,24] and individual housing [25–27] on BW, BG and Tc parameters.

We have measured cardiovascular response to BG and Tc sampling methods by HR and SAP, which are sensitive indices of stress, activated via the sympathoadrenal system [28]. Previous studies in mice subjected to Tc [29] and BG measurement [30] procedures have reported marked increases in catecholamines and corticosterone, confirming the stressful nature of these manipulations. These findings indicate that sampling methods can elicit physiological responses that may confound studies where repeat BG measurements are performed.

To minimise the influence of blood sampling on glucose tolerance test (GTT) results, a two hour recovery period following tail cut before obtaining the first blood sample has been recommended [31]. However, in our experience the wound may seal after 2 h necessitating the removal of a scab or additional cutting to collect a blood sample. Furthermore, the dose of glucose injected during GTT (2 mg/g) exerts a dominant effect over the increase in BG caused by the sampling procedure alone. Taking into consideration these findings we adopted a protocol for the GTT where the first measurement is taken immediately following tail tip excision.

To our knowledge, this is the first study to document the effects of an overnight fast on CV function and locomotor activity in group-housed mice of both sexes. We show significant increases in HR, SAP and locomotor activity in group housed females and males immediately after initiation of the fast. The magnitude and duration of these responses were similar to those recorded after cage change procedure alone, suggesting that increases in CV parameters and locomotor activity during the first 2 h of fasting are likely due to stress caused by cage transfer. In females but not in males, significantly elevated HR and locomotor activity were recorded during the dark phase and a marked reduction in SAP and HR during the final 2 h of fasting was evident. The fasting-induced attenuation in CV locomotor activity observed in females is consistent with previous observations [32] and may be due to decreased metabolic rate and activation of the parasympathetic system.

Our study adds to the literature reporting stress-like reactivity in laboratory animals caused by cage changes. Although this is well reported in rats [16,17], in mice this has been little studied [33]. In our study the magnitude of HR and SAP responses to cage change was no different from the response to more invasive procedures such as BG and Tc sampling. However, the duration of the CV changes exceeded those triggered by the sampling procedures. Long-lasting CV responses were paralleled by increased exploratory activity, believed to be triggered by the novel environment [17]. In accordance with previously published findings in rats [17], our results suggest that mice do not habituate to cage change despite the regularity of the procedure. Taking into consideration current and previous observations [17] it is recommended that a 2 h acclimatisation period is required after cage change before recording baseline CV parameters. In our phenotyping pipeline, the husbandry protocols, sequence and intervals between tests have been selected to minimise the possibility of confounding influences between tests. This is achieved by acclimatisation to the procedure room, performing cage changes within testing procedures, testing in order of perceived invasiveness and providing enough recovery time between tests.

Another important finding was the differences between sexes in physiological responses to the experimental and husbandry procedures, females showing a greater and more prolonged reduction in BW following brief individual housing, and more sustained increases in CV parameters in response to blood sampling, cage change and during overnight fast. The increase in arousal during overnight fasting was also more prolonged and pronounced in females. Consistent with our findings, Hoppe et al. 2008 [34] reported a more protracted elevation in mean arterial pressure in females after placing C57BL/6J; 129sv mice in a new metabolism cage. Greater female CV and arousal responses to experimental and husbandry procedures have also been observed in the rat [35,36]. The mechanisms underlying sex-specific differences in CV stress-responses can be explained by differences in autonomic and neuroendocrine control [37]. These findings may have important implications for the interpretation of results of phenotyping tests since it indicates that potentially confounding effects of stress are more prevalent in female mice.

In view of our results on individual housing, the increasing body of literature reporting a preference for social housing in rodents [38,39] and how this can improve welfare and aid post-operative recovery [40,41] we attempted to re-house former cage-mate mice 3 days after telemetry implantation surgery. This was previously avoided, particularly in males, due to the aggressive nature of the mouse strain in use. Applying knowledge of rodents' reliance on olfactory cues [39] and the effect of environmental enrichment [40] we designed a protocol to minimise the likelihood of aggression. Following re-grouping, active exploratory behaviour with elements of non-aggressive play-like skirmishing was observed in females. Brief instances of aggressive behaviour were seen in some males. In general it took a shorter time for females to settle and become dormant than for males (1 h vs. 2 h). Body weight data suggests social housing had a positive effect on postoperative recovery as weight gain began shortly after re-grouping. Our findings and those previously reported [41] suggest that social housing is the least stressful of housing conditions and reveals that mice can be re-housed after short term isolation and/or surgery [41].

Some of the most stressful events in husbandry result from changes in familiar cage environment. Whilst frequent cage changing is good for hygiene, it can be disruptive as rodents rely heavily on olfactory cues for recognising and communicating with cage-mates [42]. In our study an increased ammonia level was recorded in cages changed once every 14 days in comparison to those changed once every 7 days. However, none of the parameters evaluated indicated a detrimental effect of the prolonged cage-change interval. These findings are in agreement with previous studies reporting the lack of effect of cage change frequency (weekly vs. fortnightly) on ICR females [43] and C57BL/6J mice of both sexes [44] housed in IVC. Our results suggest that the changing of bedding every 14 days in IVC housing may represent a balance between maintaining good inter-cage hygiene whilst reducing the disturbance to mice.

Stress induced hyperthermia is a robust and reproducible phenomenon observed in mice and rats [21]. An increase in body temperature is elicited by removal from group housing, or by the introduction of any other stressor, such as the Tc measurement procedure itself. We have refined our working practice by using this phenomenon to assess the stress response in mice as part of our large-scale phenotyping screen by introducing a second sampling of Tc, 15 min after the initial measurement. The magnitude of Tc increase is then interpreted as a read-out for stress responsiveness, providing additional data without requiring additional animals.

## 5 Conclusion

The data presented shows that common husbandry practices and experimental procedures can have a significant impact on mouse physiology and behaviour, as evidenced by profound and sustained increases in BG, Tc, locomotor and CV activity. In general, more prolonged responses were seen during an overnight fast with cage change having an intermediate effect and sampling procedures inducing lesser and more transient responses. Procedure-induced changes in behavioural and physiological parameters were often more prolonged in females than in males (only male SAP and HR elevation in response to Tc measurement was more prolonged than the female response to the same stimuli). The stress-like responses provoked by such procedures may have a considerable influence on the outcomes of scientific studies. Ideally, for variables influenced by stress, experiments will be designed to minimise the impact of stress such that the effect of the treatment on the basal condition can be assessed. Where this is not possible, good experimental practices need to be followed with standardisation to ensure treatment and control animals are exposed to the same stressors, and randomisation to ensure hidden stressors, such as order effects, influence the results from the control and treatment animals equally. These methods will ensure that the experiment has good internal validity which in turn allows assignment of causality to the effect observed. Mathematically we cannot normalise the data for stress, as the response to stress is not linear, therefore we have to interpret the data in the presence of stress. The presence of stressors questions the external validity of the results, where external validity is the ability of the inferences to be generalised from the unique and isolated settings to other populations and conditions. Ideally, resources permitting, when a phenotype is observed the study should be repeated using an alternative technique e.g. assessment of heart rate using cardiovascular telemetry and non-invasive blood pressure. To date, the majority of animal research has inevitably been in the presence of the stressors, and we at The Sanger Mouse Genetic Project believe there is value in completing the study even with this less than ideal scenario as within our high throughput pipeline we have identified many novel phenotypes. Better insight into the reactions of laboratory mice to husbandry practice and experimental procedures will aid the refinement of animal care and standard operating protocols to avoid or minimise stress responses and improve data quality.

The following are the supplementary materials related to this article.

**Supplementary Table 1:**
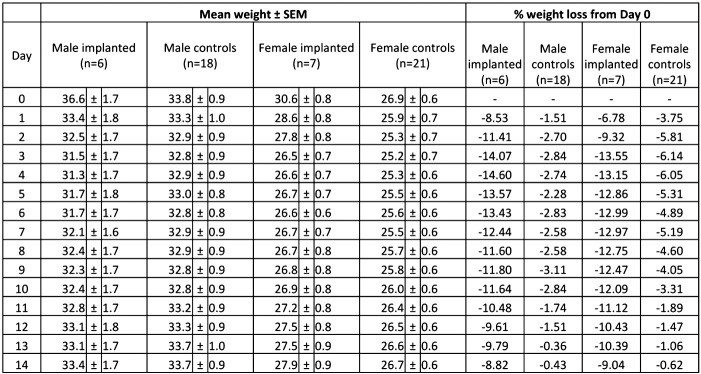
Body weights of implanted and non-implanted male and female mice. Data are expressed as mean ± SEM and as percentage change from pre-surgery or pre-separation weight (day 0). Post surgery weights were adjusted by subtraction of 1.4 g to account for the weight of the implanted transmitter.

## Figures and Tables

**Table 1:** Timing of the experimental procedures.

Age (weeks)	Procedure
17–18	Surgery
17–18 (72 h post surgery)	Re-grouping
21–22	BG sampling
22–23	Tc sampling
23–24	Ammonia measurement initiated
27–28	Overnight fast

**Table 2:** Experimental groups of non-implanted male mice.

Procedure	BG sampling	Tc sampling
T_0_ + T_15_	Group 1 (*n* = 100)	Group 9 (*n* = 10)
T_0_ + T_30_	Group 2 (*n* = 10)	Group 10 (*n* = 10)
T_0_ + T_60_	Group 3 (*n* = 10)	Group 11 (*n* = 10)
ip injection (T_0_ + T_15_)	Group 4 (*n* = 10)	Group 12 (*n* = 10)
Cage transport (T_0_ + T_60_)	Group 5, 6 (*n* = 10)	Group 13, 14 (*n* = 10)
Individual housing (T_0_ + T_60_)	Group 7, 8 (*n* = 10)	Group 15, 16 (*n* = 10)
